# Quantiferon-TB Gold in tube assay for the screening of tuberculosis before and during treatment with tumor necrosis factor alpha antagonists

**DOI:** 10.1186/ar3882

**Published:** 2012-06-18

**Authors:** Gulen Hatemi, Melike Melikoglu, Fatma Ozbakir, Koray Tascilar, Hasan Yazici

**Affiliations:** 1 Istanbul University, Cerrahpasa Medical School, Department of Internal Medicine, Division of Rheumatology, Istanbul, Turkey; 2 Istanbul University, Cerrahpasa Medical School, Central Laboratory, Istanbul, Turkey

## Abstract

**Introduction:**

The usefulness of interferon-gamma (IFN-γ) release assays for tuberculosis screening before tumor necrosis factor-alpha (TNF-α) antagonists and for monitoring during treatment is a contraversial issue. The aims of this study were to determine whether TNF-α antagonists affect the results of the Quantiferon-TB Gold in-tube assay (QTF); to assess how QTF performs in comparison with the tuberculin skin test (TST) in rheumatoid arthritis (RA) patients who are about to start treatment with TNF-α antagonists, RA patients who are not candidates for treatment with TNF-α antagonists, rheumatology patients with confirmed current or past tuberculosis infection, and healthy controls, and to determine the specificity of the QTF test to differentiate leprosy patients, another group of patients infected with mycobacteria.

**Methods:**

The 38 RA patients who were prescribed TNF-α antagonists, 40 RA patients who were not considered for TNF-α antagonist use, 30 rheumatology patients with a history or new diagnosis of tuberculosis, 23 leprosy patients, and 41 healthy controls were studied. QTF and TST were done on the same day, and both were repeated after a mean of 3.6 ± 0.2 months in patients who used TNF-α antagonists.

**Results:**

Treatment with TNF-α antagonists did not cause a significant change in the QTF or TST positivity rate (34% versus 42%; *P *= 0.64; and 24% versus 37%; *P *= 0.22). Patients with leprosy had a trend for a higher mean IFN-γ level (7.3 ± 8.0) and QTF positivity (61%) than did the other groups; however, the difference was not significant (*P *= 0.09 and *P *= 0.43).

**Conclusions:**

Treatment with TNF-α antagonists does not seem to affect the QTF test to an appreciable degree. The higher IFN-γ levels in leprosy patients deserves further attention.

## Introduction

Tuberculosis infection, usually as a reactivation of latent tuberculosis, is an important complication of treatment with tumor necrosis factor-alpha (TNF-α)-blocking drugs [1]. Guidelines have been developed in many countries for screening for latent tuberculosis before starting TNF-α antagonists [2-7]. Despite minor differences, they all include a good clinical history, physical examination, chest radiograph, and the tuberculin skin test (TST) [8].

Interpretation of the TST may be challenging for several reasons, such as false-positive results caused by Bacille Calmette-Guérin (BCG) vaccination and infection with other mycobacteria and false-negative results caused by immunosuppresssion and waning of the TST over time. An increased frequency of negative TST results has been reported in RA patients, especially among those who were eligible for TNF-α-antagonist use, probably related to disease severity, which by itself might be a cause of the immunosuppressed state [9]. Other problems with the TST are difficulties in standardization of the administration and the reading of the test.

The interferon gamma (IFN-γ) release assays (IGRAs) have emerged as promising alternatives to TST for screening latent tuberculosis. The two types of commercially available IGRAs are the Quantiferon test, which measures antigen-spesific IFN-γ released by circulating T cells in whole blood, and ELISPOT, which measures the presensitized T cells specific to *Mycobacterium tuberculosis *isolated from peripheral blood mononuclear cells, which release IFN-γ. The Quantiferon-TB gold test in-tube assay (QTF) is a newer and more practical method. The QTF test is proposed to be more sensitive and more specific than TST because it is not affected by BCG vaccination and infections with other mycobacteria [10]. However, it is hard to interpret the specificity and sensitivity of this test, as is also true for the TST, because no gold standard exists for diagnosing latent tuberculosis. It has been suggested by some that the QTF test may replace TST before starting TNF-α antagonists [11], whereas some propose that it could be used as an adjunct to the TST [12,13]. Still others suggested that it is not cost effective and reliable enough in immunosuppressed patients [14,15].

The follow-up of patients who are being treated with TNF-α antagonists for tuberculosis is a further and important problem. The duration of treatment with these agents is usually long, and many of the patients receive TNF-α antagonists for years. Apart from activation of latent tuberculosis, new tuberculosis infections can also become a problem, especially in countries with a high prevalence of tuberculosis. Guidelines are not clear on how to monitor these patients regarding the development of tuberculosis [2-4]. Thus it is important to know how these tests perform in patients receiving TNF-α antagonists.

We previously showed that the TST is not affected by treatment with TNF-α antagonists [9]. However, we did not know whether this was also true for the QTF test. A former impression suggested that the IFN-γ response may be reduced in patients who are taking TNF-α antagonists, and caution was recommended when interpreting negative IGRA results in such patients [16].

The specificity of the QTF test for differentiating *M. tuberculosis *from other mycobacteria is another issue that needs further attention. Leprosy is another mycobacterial infection that was endemic until recently in the country where this study was conducted, as well as in other countries where tuberculosis is still endemic. It was shown that the surface proteins ESAT-6 and CFP-10 used in the QTF test are also present in *M. leprae *[17]. Moreover, reactivation of leprosy was reported in three patients who were using TNF-α antagonists [18-20]. However, the QTF test had not been studied among patients with leprosy.

The aims of the current study were (a) to determine whether TNF-α antagonists affect the QTF test; (b) to determine how the QTF test performs in comparison to the TST in rheumatoid arthritis (RA) patients treated with TNF-α antagonists, RA patients who are not treated with TNF-α antagonists, rheumatology patients who had had proven tuberculosis, and healthy controls; and (c) to assess the QTF test in leprosy patients, another group of patients infected with mycobacteria.

## Materials and methods

Thirty-eight RA patients who were about to start treatment with TNF-α antagonists (TNF-RA): 40 RA patients who had not used and who were not being considered for using TNF-α antagonists (TNF-naive RA), 30 patients with various rheumatologic conditions who had a history or a new diagnosis of culture-positive tuberculosis, 23 patients with leprosy, and 41 healthy controls (HC), who were relatives or friends of patients attending our outpatient rheumatology clinic, were studied. Apart from the rheumatology patients who had had tuberculosis, none of the study subjects had a personal history or a history of contact with tuberculosis. Arms of all study subjects were checked for the presence or absence of Bacille Calmette-Guérin (BCG) scars.

Among the TNF-RA patients, 32 (84%) of 38 were using methotrexate, six (16%) of 38 were using sulfasalazine, eight (21%) of 38 were using hydroxychloroquine, 12 (32%) of 38 were using leflunomide, and 35 (92%) of 38 were using prednisolone during the first TST and QTF tests. Among TNF-naive RA patients, 31 (78%) of 40 were using methotrexate, seven (18%) of 40 were using sulfasalazine, six (15%) of 40 were using hydroxychloroquine, eight (20%) of 40 were using leflunomide, and 33 (83%) of 40 were using prednisolone during the TST and QTF tests. No significant differences were found regarding baseline treatment modalities between the two groups. The TNF-α antagonists that were used were etanercept in 16 patients (13.6 ± 0.6 weeks; range, 12 to 14), infliximab in 11 patients (three infusions at weeks 0, 2, and 6), and adalimumab in 11 patients (13.5 ± 0.7 weeks; range, 12 to 14).

After informed consent was obtained, blood was collected for the QTF assay, and TST was administered afterward. Quantiferon-TB Gold in-tube (Cellestis Ltd, Victoria, Australia) was used for the QTF assay. One milliliter of blood was drawn for each of the three test tubes. One of these tubes contained three antigens specific for *M. tuberculosis *(ESAT-6, CFP-10, and TB-7.7). The second tube was the positive control tube and was coated with the T-cell mitogen phytohemagglutinin. The third tube was the negative control tube and was coated only with EDTA. The tubes were shaken vigorously and incubated at 37°C for 24 hours. Then the tubes were centrifuged, and the supernatant was stored at -20°C until the IFN-γ was measured in an enzyme-linked immunosorbent assay (ELISA) reader. The QTF test was interpreted according to instructions of the manufacturer, by subtracting the amount of IFN-γ in the negative control tube from that in the *M. tuberculosis-*specific antigens tube. The level of IFN-γ in the negative control tube was subtracted from that in the phytohemagglutinin tube for positive control. For a positive test, all three conditions had to be met: the IFN-γ level in the negative-control well (nil) should be ≤ 8.0 IU/ml; the IFN-γ level in the well containing *M. tuberculosis*-specific antigens minus the negative control should be ≥ 0.35 IU/ml; and this same level should be ≥ 25% of nil.

TST was administered by using the Mantoux technique. The forearm was cleaned with alcohol, and 0.5 IU of purified protein derivative (PPD) solution was injected intradermally. The transverse diameter of the induration was measured 48 to 72 hours later by two independant observers. Patients put their arms through a curtain with holes to provide blinding of the observers. The hands were also covered to prevent the observers from recognizing the hand deformities of RA patients.

Patients who were prescribed TNF-α antagonists had two QTF assays and TSTs, one before the administration of the first dose of TNF-α antagonists, and the other when they came for their second prescription, after a mean of 3.6 ± 0.2 months. We think that all of our patients had reached steady-state concentrations of TNF-α antagonists by that time, because adalimumab reaches maximum steady-state concentration at 90 ± 48 hours, etanercept, at 62 ± 29 hours, and no further accumulation of infliximab was observed after the loading dose with infusions at weeks 0, 2, and 6 [21].

Mean TST indurations in each group were compared by using ANOVA. The number of patients with a positive TST and a positive QTF test in each group were compared by using the Kruskal-Wallis test. The same comparisons were made within the group of RA patients who were prescribed TNF-α antagonists, between before- and after-treatment values. The concordance between the TST and QTF tests when positive TST was defined as an induration of ≥ 10 mm was evaluated by using kappa statistics.

The study was approved by the ethical committee of Istanbul University, Cerrahpasa Medical School.

## Results

Demographic features and QTF and TST results of each group are given in Table 1. Treatment with TNF-α antagonists did not significantly affect the QTF and the TST results. The frequency of QTF positivity was 34% before starting TNF-α antagonists and 42% after 3.6 ± 0.2 months of treatment (*P *= 0.64). After treatment with TNF-α antagonists, the QTF test results did not change in 29 of 38 patients, converted from negative to positive in four of 38 patients, and reverted from positive to negative in three of 38 patiens. The two indeterminate results found before TNF-α antagonists became positive. The mean IFN-γ level in the positive-control well increased significantly (*P *< 0.001) after treatment with TNF-α antagonists, whereas the IFN-γ level in the tuberculosis-antigen well did not change (Table 2). After treatment with TNF-α antagonists, the TST positivity rate changed from 24% to 37% when a positive TST was defined as ≥ 10 mm, and from 26% to 45% when defined as ≥ 5 mm. However, the differences were not statistically significant (*P *= 0.32 and *P *= 0.15).

**Table 1:** Demographic features of patients and controls

	RA-TNF1 (*n *= 38)	RA-TNF2 (*n *= 38)	TNF-naive RA (*n *= 40)	TB (*n *= 30)	Leprosy (*n *= 23)	Healthy controls (*n *= 41)
F:M	31:7	31:7	29:11	21:9	7:16	29:12

Mean age (years)	51.7 ± 14.2	51.7 ± 14.2	52.6 ± 13.5	43.0 ± 13.4	57.1 ± 9.6	41.1 ± 12.1

BCG scar (+)	24	24	25	23	16	27

QTF						

(+)	13 (34%)	16 (42%)	20 (50%)	15 (50%)	14 (61%)	19 (47%)

(-)	23 (61%)	22 (58%)	19 (47%)	13 (43%)	8 (35%)	22 (53%)

Indeterminate	2 (5%)	0	1 (3%)	2 (7%)	1 (4%)	0

TST						

10 mm (+)	9 (24%)	14 (37%)	22 (55%)	17 (57%)	13 (57%)	28 (68%)

5 mm (+)	10 (26%)	17 (45%)	24 (60%)	21 (70%)	14 (61%)	33 (80%)

Results in rheumatoid arthritis patients before (RA-TNF1) and after (RA-TNF2) treatment with TNF-α antagonists, rheumatoid arthritis patients who were not prescribed TNF-α antagonists (TNF-naive RA), rheumatology patients who had current or past confirmed tuberculosis infection (TB), leprosy patients, and healthy controls.

**Table 2:** IFN-γ levels in the tuberculosis antigen-stimulated wells and mitogen-stimulated wells that were used as positive controls

	RA-TNF1 (*n *= 38)	RA-TNF2 (*n *= 38)	TNF-naive RA (*n *= 40)	TB (*n *= 30)	Leprosy (*n *= 23)	Healthy controls (*n *= 41)
IFN-γ level in tuberulosis antigen well (IU/ml)	3.6 ± 8.4	3.0 ± 6.2	2.8 ± 5	2.3 ± 4.2	7.3 ± 8.0	3.5 ± 6.4
IFN-γ level in positive control well (IU/ml)	7.0 ± 6.4	16.1 ± 14.2^a^	8.7 ± 6.1	11.6 ± 12.1	12.2 ± 6.1	17.3 ± 6.4

^a^The mitogen-nil level increased significantly (*P *< 0.001) after treatment with TNF-α antagonists.

The frequency of QTF positivity was lowest among TNF-RA patients before starting TNF-α antagonists (34%); however, the results were not significantly different. The frequency of TST positivity was significantly lower among TNF-RA patients before starting TNF-α antagonists (24%) compared with the rest of the groups (*P *= 0.01). The QTF positivity rate was 47% in healthy controls, whereas the TST positivity rate was 68%.

The IFN-γ levels that formed the basis for interpreting the results of the QTF test are given in Table 2. The RA-TNF1 group had the lowest positive control IFN-γ level (F_5df _= 7.58; *P *< 0.001) followed by the TNF-naive RA group (F_4df _= 5.15; *P *= 0.001).

Patients with leprosy had a higher mean IFN-γ level in the tuberculosis antigen well (7.3 ± 8.0 IU/ml) and higher QTF positivity rate (61%) than the other groups, but the difference was not significant (*P *= 0.09 and *P *= 0.43). Their TST positivity rate was not higher than that of the other groups (57%).

Among rheumatology patients who had had tuberculosis, only two had active, nontreated tuberculosis when blood was collected for the QTF test. The QTF test was positive in one of these patients and indeterminate in the other. Overall, the QTF and TST results of the rheumatology patients who had tuberculosis in the past were similar to those of the TNF-naive RA patients (50% versus 50% for QTF and 55% versus 57% for TST).

A high discordance rate was found between the QTF test and the TST (Table 3). Among the total 204 valid assays of QTF and TST, 59 (29%) were discordant, when TST positivity was defined as an induration of ≥ 10 mm (kappa = 0.41). We looked at the frequency of BCG vaccination among TST(+) QTF(-) patients to see whether this could explain the discordance in these patients. However, the number of patients with BCG scars among this group was not different from that of the rest of the patients (23 (74%) of 31, versus 119 (68%) of 173; *P *= 0.54). We had the impression that QTF(+) TST(-) patients had lower IFN-γ levels, suggesting that the low cut-off value for positivity might explain the discordance in these patients, but 10 of 28 patients who were QTF positive and TST negative had IFN-γ levels below 0.85 IU/ml.

**Table 3:** Discordance of QTF and TST results in each group, excluding patients with indeterminate QTF results

		RA-TNF1 (*n *= 36)	RA-TNF2 (*n *= 38)	TNF-naive RA (*n *= 39)	TB (*n *= 28)	LEPROSY (*n *= 22)	Healthy controls (*n *= 41)	Total (*n *= 204)
QTF (+)	TST (+)	5 (13%)	9 (24%)	15 (38%)	12 (40%)	12 (52%)	16 (39%)	69 (34%)
	TST (-)	8 (21%)	7 (18%)	5 (13%)	3 (10%)	2 (9%)	3 (7%)	28 (14%)
QTF (-)	TST (+)	4 (11%)	5 (13%)	6 (15%)	3 (10%)	1 (4%)	12 (29%)	31 (15%)
	TST (-)	19 (50%)	17 (45%)	13 (33%)	10 (33%)	7 (30%)	10 (24%)	76 (36%)

Tuberculin skin test (TST) positivity is defined as an induration of ≥ 10 mm.

Interobserver reliability for reading the TST was good (kappa = 0.95). The interobserver reliability of the same two observers was similar in our previous study evaluating TST in patients who were using infliximab (kappa = 0.92) [9].

## Discussion

This study suggested that treatment with TNF-α antagonists does not affect the QTF test. The QTF test was positive in 34% of the patients before treatment and in 42%, 3.6 months later. It was previously shown that IFN-γ production in response to phytohemagglutinin is impaired in RA patients and is more pronounced in RA patients with high inflammatory activity compared with those with low activity [22,23]. The fact that the IFN-γ level in the positive-control well increased significantly after treatment with TNF-α antagonists, probably because of an improvement in the immunocompromised state of these patients, whereas the IFN-γ level in the tuberculosis-antigen well did not change, also supports the notion that results of the QTF test are not affected by treatment with TNF-α antagonists. This is important for showing the utility of this test during treatment with TNF-α antagonists.

Several mechanisms are thought to play roles in the impairment of immunity against tuberculosis in patients using TNF-α antagonists [24]. One explanation would be that these drugs interfere with the secretion of IFN-γ by memory T cells [24]. This raises concern that IGRAs may not be useful in patients who are already using TNF-α antagonists. An *ex vivo *study of patients treated with TNF-α antagonists showed that the number of IFN-γ-releasing lymphocytes and immediate release of IFN-γ after challenging with mycobacterial antigens was significantly decreased after 14 weeks of treatment with these drugs, compared with the initial values [25]. The authors concluded that ELISPOT assays are not reliable to diagnose previous or latent tuberculosis in patients using TNF-α antagonists. However, and in line with what we observed here, a clinical study with serial ELISPOT testing before and after treatment with infliximab suggested that ELISPOT is a reliable tool for monitoring tuberculosis in these patients [26]. Similarly, a cross-sectional study that looked at the QTF-Tb Gold test in patients who were already using TNF-α antagonists showed that the number of patients with a positive test was lower in the group treated with TNF-α antagonists and suggested that QTF-Tb Gold positivity decreased with TNF-α antagonist use [27]. However, QTF-Tb Gold assays were not available before treatments with TNF-α antagonists were started in these patients. In contrast, our results provide evidence that the QTF-Tb Gold test is not affected by TNF-α antagonists. Thus it is important to test how IGRAs perform in different immunosuppressed patient populations with serial testing before and after treatment with these agents, especially in settings with a high tuberculosis prevalence.

The lower QTF and TST positivity among RA patients who are prescribed TNF-α antagonists, compared with those who are not, is probably related to the higher disease activity in the first group. The demographic features and the drugs used when the first QTF test was performed were similar between the two groups. However, the first group had a higher disease activity, causing them to be prescribed TNF-α antagonists. A formal analysis of disease activity was not performed for the purposes of this study, but a minimum DAS28 score of 5.1 is required for prescribing TNF-α antagonists in Turkey.

Our study showed that the positivity rate of QTF was higher than the TST among RA patients and lower than the TST in healthy controls. However, because no gold standard exists for diagnosing latent tuberculosis, it is impossible to know definitely which test is more accurate. The QTF test was positive in 47% of healthy controls, and the TST was positive in 68%. The estimated prevalence of latent tuberculosis, defined as TST positivity among those who did not have a BCG vaccination, is 25% in Turkey [28]. Thus, assuming that our healthy controls reflect the genreal population in Turkey, we can speculate that the false-positivity rate of QTF may be lower than that of TST, but it is probably still quite high.

Patients with leprosy had the highest QTF positivity rate and mean IFN-γ levels in tuberculosis-antigen wells among the groups we studied, whereas their IFN-γ levels in positive-control wells and TST positivity rate were not higher than those in the other groups. This finding suggests that the QTF test may give false-positive results in leprosy patients because of cross-sensitivity with *M. tuberculosis *antigens. As far as we know, this is the first study to look at the QTF test in leprosy patients. It may be true that patients with leprosy live in worse socioeconomic conditions, spend more time in hospitals and institutions, and may be more exposed to *M. tuberculosis*. However, none of the patients we studied had a previous personal history or history of contact with *M. tuberculosis*. Thus it is possible that infection with *M. leprae *might be causing positive QTF results.

The discordance rate between the QTF test and the TST was 29% in our study. In another study from Turkey, which looked at the agreement between QTF and TST results among RA and AS patients, the QTF was positive among 35%, the TST was positive among 66% of the patients, and the discordance rate was 39% [29]. Another study from Turkey, this time among health-care workers, showed a discordance rate of 37% [30]. The discordance rate is usually lower in reports from countries where tuberculosis prevalence is low, because most patients are both QTF and TST negative [31]. In countries where tuberculosis is endemic, discordance rates are usually higher [32]. It is suggested that BCG vaccination is one of the reasons for discordance when the TST is positive and the QTF is negative. However, in our population, this was not the case. The number of patients with a BCG scar was similar among TST(+) QTF(-) patients and the rest of the patients. Conversely, 14% of our patients were QTF(+) and TST(-). These patients usually had low IFN-γ levels. This suggests that the current low cut-off value for QTF positivity, as advised by the manufacturer, may be reponsible for some of the QTF(+)TST(-) results, thus increasing the sensitivity, but decreasing the specificity of the test. In our study population, increasing the cut-off for QTF positivity to 0.85 IU/ml would decrease the frequency of QTF(+)TST(-) discordant results from 14% to 9%. The problem with the cut-off value was emphasized by another study, which showed that reproducibility is low among patients with IFN-γ levels close to the cut-off point [33]. Among patients with inconsistent results between two consecutive tests, maximum IFN-γ response in either test was 0.68 IU/ml, compared with 4.99 IU/ml in persistently positive patients [33]. Thus, it is important to interpret QTF results, not just as negative or positive, but quantitatively, together with IFN-γ levels in the positive-control and tuberculosis-antigen wells, especially in immunosuppressed populations.

We had only two rheumatology patients with active tuberculosis at the time of this study. The other patients who had had tuberculosis in the past had received sufficient antituberculosis treatment. This may be the reason for the low QTF positivity in this group. However, QTF positivity rates of as low as 64% have previously been reported among patients with active tuberculosis [34].

The main limitation of our study is the same with all QTF and, for that matter, TST studies. No gold standard exists for diagnosing latent tuberculosis. In most studies about the sensitivity of the QTF test for latent tuberculosis, surrogates (such as history of exposure to a patient with tuberculosis, sequelae on chest radiograph, or active tuberculosis) have been used [10]. Because of this uncertainty, we did not attempt to calculate sensitivities and specificities of the QTF and TST tests. We compared the results of patient groups with healthy controls instead.

The other limitation of our study was that it lacked long-term follow-up. Although the prospective nature of our study among patients using TNF-α antagonists gave us an idea about the effect of anti-TNF treatment on the QTF test, we do not know whether being QTF positive shows a risk for activation of latent tuberculosis in the long term. In our limited experience, we can point out that among the eight patients who were QTF positive and TST negative initially, and who did not use isoniazid, none has developed tuberculosis. It is reasonable to postulate that these same patients would be expected to develop activation within the first few months of TNF-α antagonists use, had they had latent tuberculosis. However, data on more patients are needed to comment on the safety of not treating such patients, as well as patients who had QTF conversion from negative to positive during treatment with TNF-α antagonists.

When considering QTF as a means for screening latent tuberculosis in large populations, it has advantages, such as the lack of boosting and no need for return visits, but it also has several problems. It is expensive and requires specialized laboratories. Its positivity rate in other mycobacteria and its reproducibility and reliability must be studied further in immunocompromised populations.

Previous studies comparing QTF and TST for screening latent tuberculosis before starting TNF-α antagonists suggested that these tests show good concordance in populations with low tuberculosis prevalence [35]. In such settings, the QTF test may be used either to confirm positive TST results or when false-negative TST results are suspected [12].

## Conclusions

In conclusion, the QTF test does not seem to be affected by TNF-α antagonists. In terms of screening patients for latent tuberculosis before starting TNF-α antagonists, no convincing data favor the QTF test or the TST. Moreover, considering feasibility and cost-effectiveness, it is hard to recommend the routine use of QTF test for screening latent tuberculosis before treatment with TNF-α antagonists in countries with a high prevalence of tuberculosis. The frequency of reactivation of tuberculosis during anti-TNF therapy has significantly decreased with routine screening before starting treatment [36]. However, this decrease seems to be related to increased clinical and radiologic awareness rather than to the QTF or the TST. This is further supported by the observation that, in a large series of validated tuberculosis cases among patients using TNF-α antagonists, initial TST was negative in 67% of the patients [37].

## Abbreviations

BCG: Bacille Calmette-Guérin; HC: healthy control; IFN-γ: interferon gamma; IGRA: interferon gamma release assay; QTF: Quantiferon-TB Gold in-tube assay; RA: rheumatoid arthritis; TNF-α: tumor necrosis factor alpha; TNF-naive RA: RA patients who had not used and who were not being considered for using TNF-α antagonists; TNF-RA: RA patients who were about to start treatment with TNF-α antagonists; TST: tuberculin skin test.

## Competing interests

The authors declare that they have no competing interests.

## Authors' contributions

GH contributed in designing the study, collected and analyzed data, and drafted the manuscript. MM contributed in designing the study, collection of data, and drafting of the manuscript. FB performed the laboratory analyses. KT contributed in collection of data. HY contributed in designing the study, analysis of data, and drafting of the manuscript. All authors read and approved the manuscript.
